# A correlation of the adsorption capacity of perovskite/biochar composite with the metal ion characteristics

**DOI:** 10.1038/s41598-023-36592-5

**Published:** 2023-06-10

**Authors:** Shimaa M. Ali, Mohamed A. El Mansop, Ahmed Galal, Soha M. Abd El Wahab, Wafaa M. T. El-Etr, Hanaa A. Zein El-Abdeen

**Affiliations:** 1 Chemistry Department, Faculty of Science, Cairo University, Giza, 12613 Egypt; 2 Physics Department, Faculty of Science, Cairo University, Giza, 12613 Egypt; 3 Soil, Water and Environmental Research Institute, Agriculture Research Center (ARC), Giza, 12613 Egypt

**Keywords:** Chemistry, Materials science

## Abstract

LaFeO_3_/biochar composite is prepared by cellulose-modified microwave-assisted method at 450 °C. The structure is identified by Raman spectrum which, consists of characteristics biochar bands and octahedral perovskite chemical shifts. The morphology is examined by scanning electron microscope (SEM); two phases are observed, rough microporous biochar and orthorhombic perovskite particles. The BET surface area of the composite is 57.63 m^2^/g. The prepared composite is applied as a sorbent for the removal of Pb^2+^, Cd^2+^, and Cu^2+^ ions from aqueous solutions and wastewater. The adsorption ability reaches a maximum at pH > 6 for Cd^2+^, and Cu^2+^ ions, and is pH-independent for Pb^2+^ ions adsorption. The adsorption follows pseudo 2nd order kinetic model, Langmuir isotherm for Pb^2+^ ions, and Temkin isotherms for Cd^2+^, and Cu^2+^ ions. The maximum adsorption capacities, *q*_*m*_, are 606, 391, and 112 mg/g for Pb^2+^, Cd^2+^, and Cu^2+^ ions, respectively. The electrostatic interaction is responsible for the adsorption of Cd^2+^, and Cu^2+^ ions on LaFeO_3_/biochar composite. In case of Pb^2+^ ions form a complex with the surface functional groups of the adsorbate. LaFeO_3_/biochar composite shows high selectivity for the studied metal ions and excellent performance in real samples. The proposed sorbent can be easily regenerated and effectively reused.

## Introduction

Water pollution is one of the critical problems facing humanity, especially with increasing the worlds’ population and the need for more industrial and agriculture activities, fossil fuel utilization, domestic sewage, mining, and urbanization^1^. Heavy metal ions, such as Pb^2+^, Cd^2+^, and Cu^2+^ ions constitute the most dangerous water pollutants due to its toxicity even in low levels, non-degradability, and bioaccumulation^2^. It is not only affecting the aquatic life, but also can affect human health. Pb^2+^ ions can cause kidney and liver damage and reduced fertility^3^, Cd^2+^ ions cause renal disorder, human carcinogen^4^, and Cu^2+^ ions cause Wilson disease, insomnia^5^. According to the Unites States Environmental Protection Agency (US EPA), the maximum limits of Pb^2+^, Cd^2+^, and Cu^2+^ ions should not exceed 0.01, 0.005, and 1.3 mg/l, respectively^6^. Several methods exist for the removal of heavy metal ions, such as coagulation^7^, flocculation^8^, ion-exchange^9^, chemical precipitation^10^, membrane filtration^11^, ultrafiltration, nanofiltration^12^, flotation, reverse osmosis^13^, electro dialysis^14^, photocatalysis^15^, and microwave catalysis^16,17^. However, these methods are complicated and expensive^18^. Adsorption method is simple, cost-effective, and with the recent development of sorbent materials, it becomes highly efficient. Sorbents can be regenerated and reused several times with maintained efficiency^19–23^.

Perovskites can be prepared in the nanometer range and have the general formula ABO_3_, where A is a rare earth, alkali, or alkaline earth metal and B is a transition metal^24^. Perovskites can be used in many important applications such as catalysis, sensors, energy production and storage, optical and electronic devices^25–28^, etc. due to their outstanding magnetic, electrical, optical, surface and structural properties^29–32^. The use of perovskites as sorbent materials is reported^33,34^, for examples, for the removal of Pb^2+^ and Cd^2+^ ions by CaTiO_3_ nanospheres^35^, and by LaGdO_3_^36^. The removal of heavy metal ions by XFe_2_O_4_-BiFeO_3_ (X = Cr, Mn, Fe, Co, or Ni)^37^, the removal of Cd^2+^ ions by LaFeO_3_^38^, and Sr^2+^ ions by BaTiO_3_^39^. All these studies show that perovskites can be used effectively as sorbents for the removal of toxic heavy metal ions, and the adsorption performance can be controlled by the perovskite structure and the synthesis method.

Biochar is a porous carbon-rich solid material that can produced by the heating the biomass in an oxygen-limited environment^40^. Biochar can be prepared by the pyrolysis of agricultural wastes, wood materials, sludge, animal residue, fruit peel, and other wastes^41^. Due to the low-cost, high surface area, and high cation capacity, biochar can be used effectively for the removal of toxic heavy metal ions by adsorption^42^. The removal mechanism involves physical adsorption, electrostatic interactions, metal ion-π interactions, and/or complexation^41,43^. Biochar is used for the removal of Cr^6+^, As^5+^, Cu^2+^, and Pb^2+^ ions^41^. However, expensive separation techniques are required, due to the small particles size of the biochar^44^. It is recommended to use biochar composites with magnetic materials to enhance the sorbent^43^.

Few papers report the adsorption performance of perovskites and their composites for toxic heavy metal ions. Biochar is expected to effectively and economically enhance the sorption ability of perovskites by composite formation. To our knowledge, it is the first time to use perovskite/biochar composite for the removal of heavy metal ions. In this work, LaFeO_3_/biochar composite is prepared by the cellulose-modified microwave-assisted citrate method at relatively low calcination temperature, 450 °C. The sorption performance of the prepared composite for Pb^2+^, Cd^2+^, and Cu^2+^ ions is investigated in aqueous solutions and wastewater. Factors affecting the adsorption process such as, pH, contact time, and initial metal ion concentration are studied. A kinetic study and adsorption isotherms are discussed, and the adsorption capacities for different metal ions are determined and correlated to the metal ion characteristic. Finally, the selectivity and the possibility of the sorbent regeneration are examined.

## Results and discussion

### Characterization of LaFeO_3_/biochar composite

#### Raman spectroscopy

**Figure 1:**
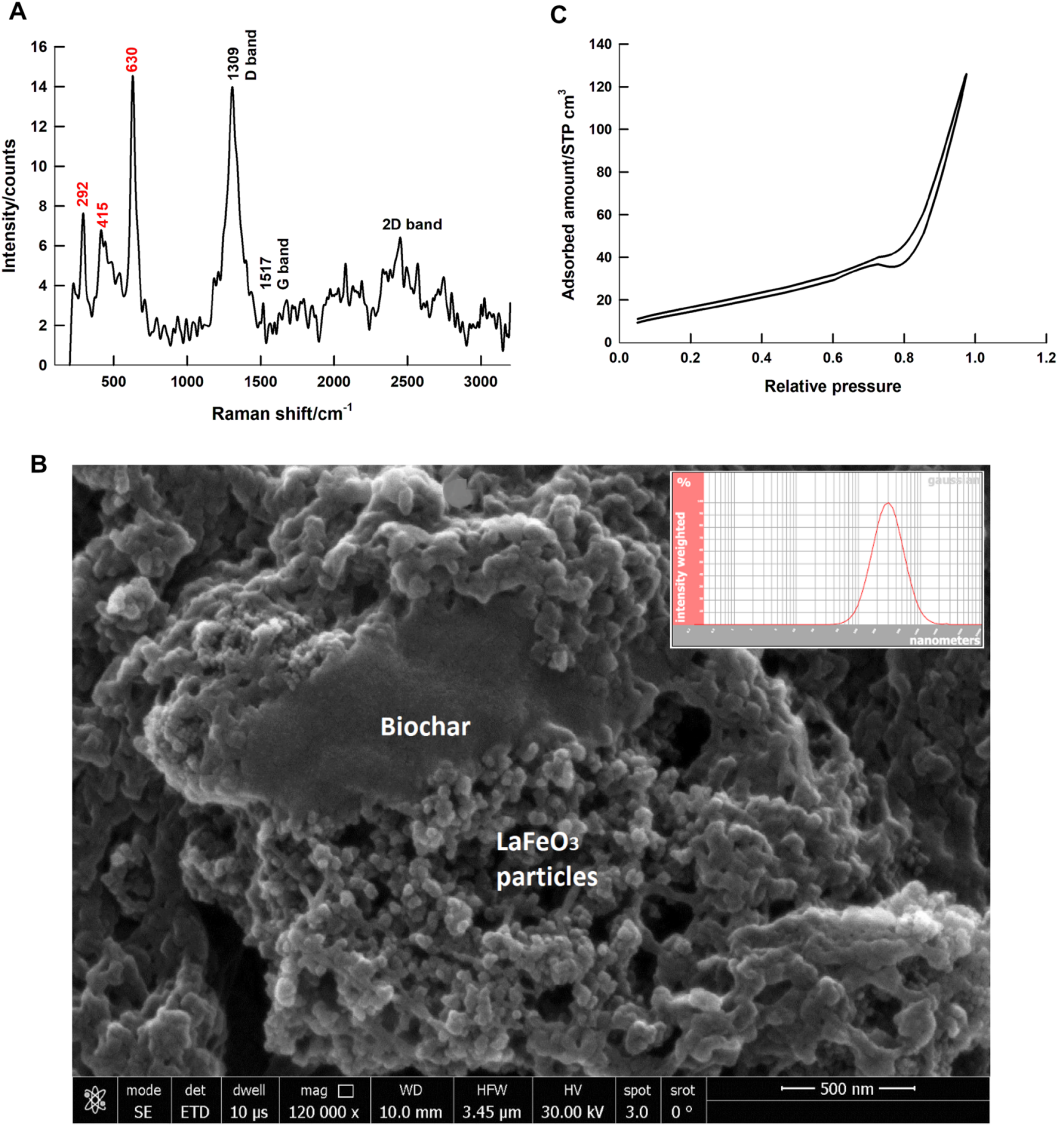
Raman spectrum (**A**), SEM image (**B**) of LaFeO_3_/biochar composite, prepared by the microwave-assisted method at 450 °C, the inset is the particle size distribution, BET hysteresis loop (**C**).

Identification of the structure is confirmed by the analysis of the Raman spectrum of LaFeO_3_/biochar composite, prepared by the microwave-assisted citrate method at relatively low calcination temperature of 450 °C, shown in Fig. 1A. It is reported that Raman spectrum of biochar, prepared from BP, consists of three main bands; the band G at 1567 cm^−1^, the band D at 1372 cm^−1^, and the band 2D which is extended between 2400 and 3300 cm^−1^ ^45^. The G band corresponds to E2g mode of hexagonal graphite, and its position indicates the extent of the charge transfer. While, the D band corresponds to sp2 pattern of a carbon ring, it is active only in the presence of structure defects. The Raman spectrum of LaFeO_3_/biochar composite shows a low intensity G band and a high intensity D band with a lower wavelength Raman shifts for both bands. This indicates that the composite formation with LaFeO_3_ perovskite affects the biochar structure and largely increases the structure defects. On the other hand, characteristic Raman bands for LaFeO_3_ perovskite are located at 292 and 415 cm^−1^ for oxygen octahedral tilt and bending vibration, respectively, 630 cm^−1^ for oxygen stretching vibration, and at 1320–1350 cm^−1^ for the scattering of oxygen vibrations, which interferes with the D band of the biochar^46^.

#### Surface characterizations

The surface morphology of LaFeO_3_/biochar composite, prepared by the microwave-assisted method at 450 °C, is examined by SEM. Figure 1B shows SEM image of LaFeO_3_/Biochar Composite, two phases can be noticed: a rough rigid microporous phase of the biochar, and orthorhombic nanoparticles of LaFeO_3_ perovskite. The composite is highly porous, and contains pores and slits of different sizes. The inset represents the particle size distribution, determined by DLS method, the average particle diameter is 355 nm.

Figure 1C shows the BET hysteresis loop, which is H3 type, according to IUPAC guidelines. Therefore, the composite contains slit-shaped pore and panel-shaped particles^47^, which agrees well with the morphological features observed in SEM image, Fig. 1B. The calculated BET surface area is 57.63 m^2^/g, and the pore volume and size are 0.1953 cc/g and 6.78 nm, respectively. It is worth to mention that**,** the surface area of LaFeO_3_ is largely increased from 6.7 m^2^/g^29^ to 57.6 m^2^/g by forming a composite with the biochar.

### Application of LaFeO_3_/biochar composite as a sorbent for the removal of Pb(II), Cd(II), and Cu(II) ions from aqueous solutions

#### Effect of pH

**Figure 2:**
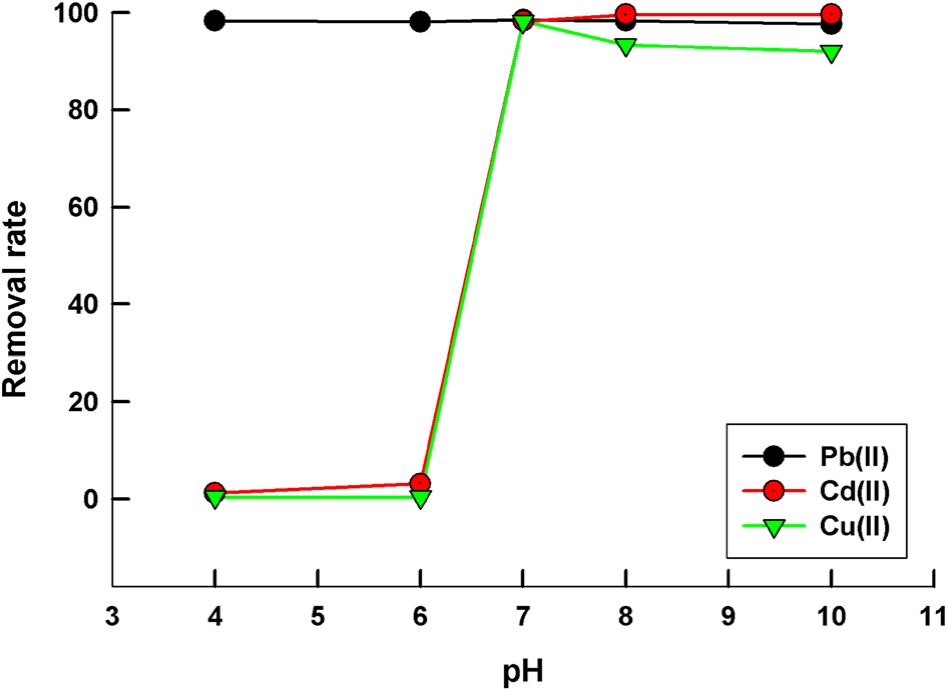
The dependence of the removal rate of Pb^2+^, Cd^2+^, and Cu^2+^ ions by LaFeO_3_/biochar composite on the pH, initial metal ion concentration = 30 ppm, contact time = 24 h, at room temperature.

Studying the effect of pH is important in order to optimize the adsorption process and to predict the possible mechanism of the adsorbate-sorbent interaction. In this work, the studied pH range is varied from 4 to 10, Fig. 2 shows the dependence of the removal rate with the pH of the solution for the adsorption of different metal ions (Pb^2+^, Cd^2+^, and Cu^2+^) on LaFeO_3_/biochar composite. The removal rate is high at pH value ≥ 7, 98.5, 98.3, and 98.1% for Pb^2+^, Cd^2+^, and Cu^2+^ ions, respectively at pH = 7. On the other hand, the removal rate is close to zero for Cd^2+^, and Cu^2+^ ions at pH value < 7, while for Pb^2+^ ions, high removal ability is still preserved.

The point of zero charge, pH_PZC_, is the pH value at which the sorbent surface is neutral. It is reported that the pH_PZC_ of perovskite is 5^33^, while that of the biochar is 7^40^. For the proposed LaFeO_3_/biochar composite, the surface charge is negative at pH value > 6. The removal rate is high at pH > 6 due to the electrostatic attraction between the negatively charged sorbent and the positively charged metal ions as well as the positively charged hydrolysis products such as M(OH)^+^, M_2_(OH)^3+^, M is Pb^2+^, Cd^2+^, or Cu^2+^ ions^48^. The formation of negatively charged hydrolysis products or even metal hydroxides can be achieved at higher pH values, pH > 10. For this reason, the pH range examined in this work is limited to pH = 10. At lower pH values < 7, LaFeO_3_/biochar composite is positively charged thus, its particles repel with the positively charged metal ions and no adsorption occurs. It can be noticed that the sorption ability remains high at pH < 7 for Pb^2+^ ions only, suggesting the presence of other type of interactions, other than the electrostatic interaction, between Pb^2+^ ions and perovskite/biochar material^49^.

#### Kinetic study

**Figure 3:**
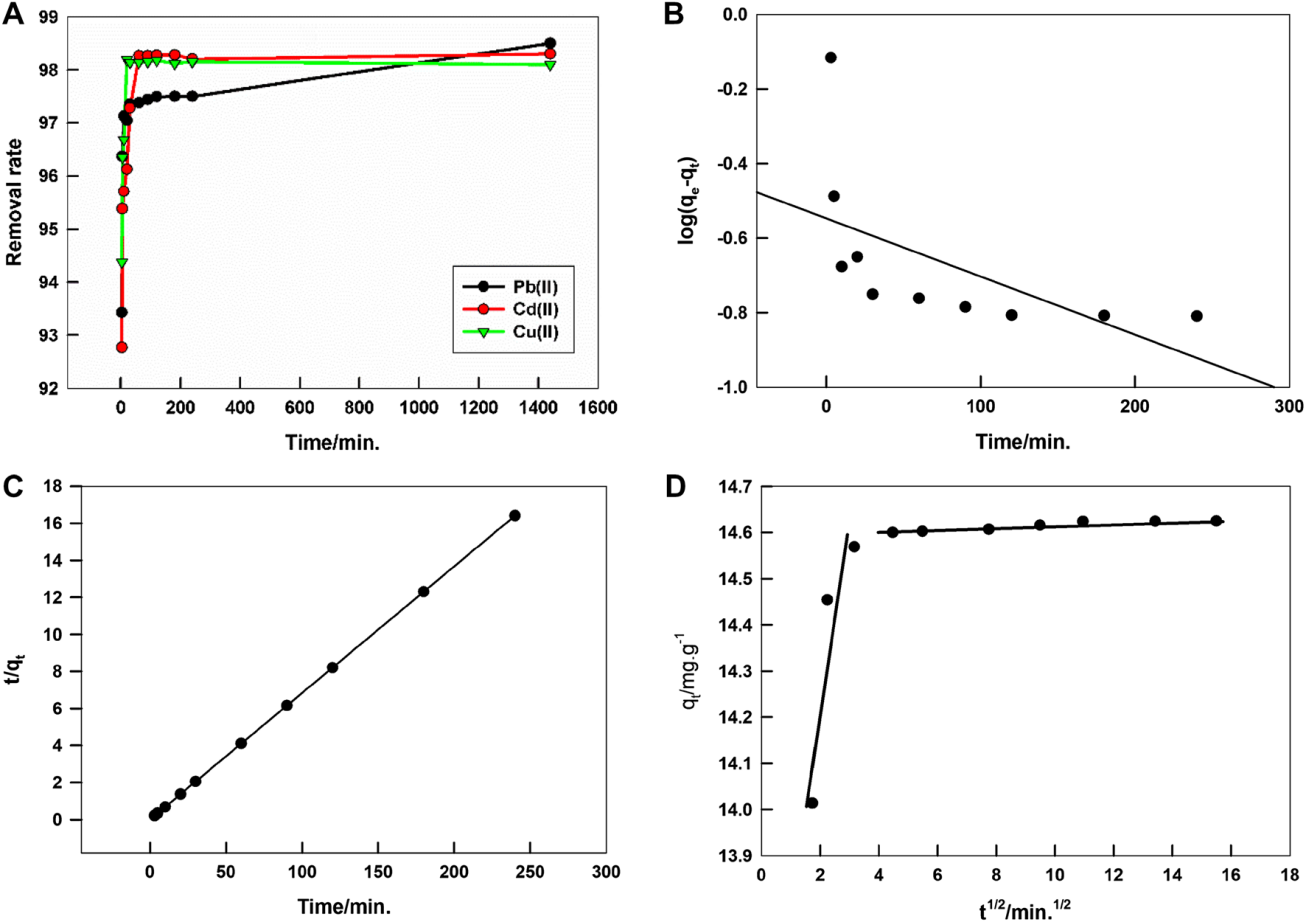
The variation of the removal rate of Pb^2+^, Cd^2+^, and Cu^2+^ ions by LaFeO_3_/biochar composite, initial metal ion concentration = 30 ppm, pH = 7, at room temperature (**A**). Pseudo 1st order (**B**), pseudo 2nd order (**C**), and Intra-particle diffusion (**D**) kinetic models for the adsorption of Pb^2+^ ions.

A kinetic study is carried out by performing the adsorption experiment at different contact times, starting from few minutes to 24 h. Figure 3A shows the variation of the removal rate of Pb^2+^, Cd^2+^, and Cu^2+^ ions by LaFeO_3_/biochar composite with the adsorbate-sorbent contact time while shaking the solution. In the first interval of time, the adsorption rate increases rapidly due to the adsorption of the metal ions on the outer adsorption sites of the sorbent; adsorption equilibrium is achieved after 30 min. It is worth to mention that the adsorption removal rate values attained at the end of the first few minutes, are close to the equilibrium value, which reflects the successful and quick removal ability of the proposed sorbent and make it highly recommended for the field applications.

In order to gain more information about the adsorption mechanism and the sorption characteristics, pseudo-first and -second order models are applied to the kinetic data.

According to pseudo-1st and -2nd order kinetics models, given by Eqs. (1) and (2)^50^:

1 $$\mathit{log}({q}_{e}-{q}_{t})=\mathit{log}{q}_{e}-\frac{{k}_{1}}{2.303}t$$

2 $$\frac{t}{{q}_{t}}=\frac{1}{{k}_{2}{q}_{e}}+\frac{t}{{q}_{e}}$$

where *q*_*e*_ and *q*_*t*_ are adsorbed amount of the pollutant metal ion at equilibrium and at time, *t* (mg/g), *k*_*1*_ and *k*_*2*_ are pseudo 1st and 2nd order rate constant, respectively.

Figure 3B and 3C represent the 1st order and 2nd order models for the adsorption of Pb^2+^ ions by LaFeO_3_/biochar composite, kinetics models for the adsorption of Cd^2+^, and Cu^2+^ ions are shown in supplementary, Figure S1. According to the correlation coefficient values, *R*^2^, pseudo 2nd order model fits better the kinetic data for all studied metal ions, as shown in Table 1. Also, the experimental *q*_*e*_ values show a better agreement with calculated *q*_*e*_ values by pseudo 2nd order model than by pseudo 1st order model. Therefore, the adsorption of Pb^2+^, Cd^2+^, and Cu^2+^ ions by LaFeO_3_/biochar composite follows 2nd order rather than 1st order kinetic model, indicating the chemisorption mode of the removal process^51,52^.

**Table 1:** Calculated kinetics parameters from fitting of pseudo 1st and 2nd order and intra-particle diffusion models on the adsorption data of Pb^2+^, Cd^2+^, and Cu^2+^ ions by LaFeO_3_/biochar composite.

Metal ion	*q*_*e,exp*_ (mg/g)	Pseudo 1st order model			Pseudo 2nd order model		
		*q*_*e,cal*_ (mg/g)	*k*_*1*_ (min^−1^)	*R*^2^	*q*_*e,cal*_ (mg/g)	*k*_*2*_ (mg/g min)	*R*^2^
Pb(II)	14.78	0.28	0.0036	0.3434	14.71	12.59	0.9999
Cd(II)	14.90	0.40	0.0043	0.1859	14.62	4.50	0.9999
Cu(II)	14.83	0.15	0.0018	0.0242	14.61	4.18	0.9999

Intra-particle diffusion model							
Metal ion		Surface diffusion			Pore diffusion		
		*k*_*id*_ mg/g min^½^	*C*_*i*_	*R*^2^	*k*_*id*_ mg/g.min^½^	*C*_*i*_	*R*^2^
Pb(II)		0.36	13.50	0.9774	2.46 $\times$ 10^−3^	14.59	0.9854
Cd(II)		0.08	14.10	0.9899	9.58 $\times$ 10^−3^	14.72	0.9928
Cu(II)		0.15	14.02	0.9863	6.72 $\times$ 10^−3^	14.64	0.9409

The adsorption occurs on the outer active sites of the sorbent with the possible diffusion of ions into the pores of the sorbent, which may additionally affect the adsorption mechanism. According to Weber and Morris model, a linear relation is expected between *q*_*t*_ and the square root of time, *t*^0.5^, and the intra-particle diffusion rate can be calculated by^52^:

3 $${q}_{t}={k}_{id}{t}^{0.5}+{C}_{i}$$

where *k*_*id*_ is the intra-particle diffusion rate constant, and *C*_*i*_ is the intercept. In case of zero intercept value, the intra-particle diffusion is the rate-controlling step.

Figure 3D shows the intra-particle diffusion model for the adsorption of Pb^2+^ ions by LaFeO_3_/biochar composite. The curve consists of two line-regions, which indicates that two mechanisms affect the adsorption process. The two mechanisms are diffusion of the adsorbate species on the surface and to the pores of the sorbent as represented by the first and the second lines, respectively. Values of *k*_*id*_ and intercepts of the first and the second lines for the adsorption of Pb^2+^, Cd^2+^, and Cu^2+^ ions on LaFeO_3_/biochar composite are calculated and listed in Table 1. It can be noticed that intercepts of the first and the second lines are comparable for each metal ion, which reflects that the surface and pore diffusion steps contribute equally to the rate-determining step of the adsorption^52^.

#### Effect of the initial metal ion concentration

**Figure 4:**
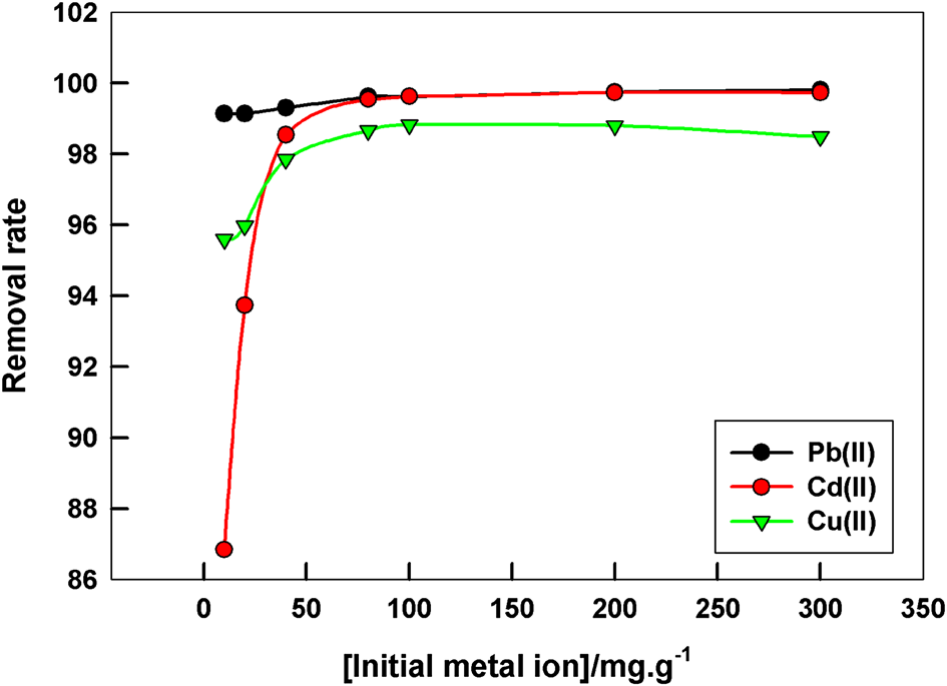
The dependence of the removal rate of Pb^2+^, Cd^2+^, and Cu^2+^ ions by LaFeO_3_/biochar composite on the initial metal ion concentration, pH = 7, contact time = 2 h, at room temperature.

The dependence of the sorption ability of LaFeO_3_/biochar composite on the initial pollutant metal ion concentration is investigated. Figure 4 shows the variation of the removal rate of Pb^2+^, Cd^2+^, and Cu^2+^ ions by LaFeO_3_/biochar composite. The removal rate has a high and a constant value at any initial concentration, for Pb^2+^ and Cu^2+^ ions. While, for Cd^2+^ ions, it increases with increasing the initial concentration and then reaches a constant value. This reflects the excellent adsorption ability of the proposed sorbent at all pollutant within the studied concentration range.

##### Adsorption isotherms

In order to elucidate a better understanding of the adsorption mechanism, the adsorption data are fitted to Langmuir, Freundlich, and Temkin isotherms. Langmuir isotherm assumes a monolayer adsorption of the adsorbate species on homogeneous and identical adsorption sites of the sorbent with no lateral interactions. While, Freundlich isotherm assumes both monolayer and multilayer adsorption on heterogeneous adsorption sites with no lateral interaction. Temkin isotherm postulates that there is an adsorbate-sorbent interaction.

Linear forms of these isotherms are given by the Eqs. (4), (5), and (6), respectively^53^:

4 $$\frac{1}{{q}_{e}}=\frac{1}{{q}_{m}}+\frac{1}{{q}_{m} {K}_{L}{C}_{e}}$$

5 $$\mathit{ln}{q}_{e}=\mathit{ln}{K}_{F}+\frac{1}{n}\mathit{ln}{C}_{e}$$

6 $${q}_{e}=\frac{RT}{b}ln{K}_{T}+ \frac{RT}{b}\mathrm{ln}{C}_{e}$$

where *q*_*m*_ is the maximum adsorption capacity (mg/g), *K*_*L*_ is Langmuir constant which is related to the adsorption free energy. *K*_*F*_ and *n* are Freundlich constants related to the adsorption capacity and the heterogeneity of the adsorption sites. *R* is the universal gas constant, *T* is the absolute temperature, *b* is Temkin constant which is related to the heat of adsorption, and KT is the equilibrium binding constant.

The adsorption data of Pb^2+^, Cd^2+^, and Cu^2+^ ions on LaFeO_3_/biochar composite are fitted to different isotherms as shown in supplementary, Figure S2. Adsorption parameters are calculated and listed in Table 2. According to the correlation coefficient, *R*^2^, it can be concluded that the adsorption of Cd^2+^, and Cu^2+^ ions on LaFeO_3_/biochar composite follows Temkin isotherm, while the adsorption of Pb^2+^ ions on LaFeO_3_/biochar composite follows Langmuir isotherm. A strong electrostatic interaction is developed between the positively charged Cd^2+^ and Cu^2+^ ions and LaFeO_3_/biochar composite, which facilitates the adsorption ability at pH > 6 (the sorbent is negatively charged), and retards it at pH ≤ 6 (the sorbent is positively charged), this trend is shown in Sect. 2.2.1. Therefore, the adsorption data of Cd^2+^ and Cu^2+^ ions by LaFeO_3_/biochar composite fits best Temkin isotherm which, assumes the presence of an interaction between the adsorbed metal ions and the sorbent. On the other hand, the adsorption of Pb^2+^ ions on LaFeO_3_/biochar composite, shows a unique pH-independent behavior. As the adsorption involves a complex formation between the adsorbed Pb^2+^ ions and the functional groups on the sorbent surface, rather than electrostatic interaction. Thus, the adsorption data for Pb^2+^ ions on LaFeO_3_/biochar composite fits best Langmuir isotherm.

**Table 2:** Adsorption parameters calculated by fitting Langmuir, Freundlich, and Temkin isotherms to the adsorption data of Pb^2+^, Cd^2+^, and Cu^2+^ ions on LaFeO_3_/biochar composite.

Metal ion	Langmuir isotherm			Freundlich isotherm			Temkin isotherm		
	q_m_ (mg/g)	K_L_ (L/mg)	R^2^	n	K_F_ (mg^1−(1/n)^ L^1/n^/g)	R^2^	b (kJ/mol)	K_T_ (L/mg)	R^2^
Pb(II)	**606.06**	**0.13**	**0.9946**	**0.86**	106.70	0.8722	42.47	16.95	0.6347
Cd(II)	111.86	0.75	0.8986	0.63	225.88	0.9347	**332.92**	**3.78**	**0.9886**
Cu(II)	390.63	0.11	0.9532	1.54	43.82	0.9149	**134.03**	**2.14**	**0.9788**

Significant values are in [bold].

Based on the maximum adsorption capacity values, *q*_*m*_, calculated from Langmuir isotherm, LaFeO_3_/biochar composite has the following adsorption ability order: Pb^2+^  > Cu^2+^  > Cd^2+^ ions with *q*_*m*_ values of 606.06, 390.63, and 111.86 mg/g, respectively. This order can be explained on the basis of hard-soft-acid–base (HSAB) principle, which divides metal ions into hard, soft, and borderline ions according to the ligand type that can form the most stable complex with these ions^54,55^. HSAB principle has been used to describe the adsorption of some metal ions by complex formation with functional groups at the sorbent surface^54^. An experimental and theoretical study on the adsorption of some metal ions on graphene oxide, shows that the order of *q*_*m*_ is: Na^+^  < Mg^2+^  < Co^2+^  < **Cd**^**2+**^  < Zn^2+^  < Ni^2+^  < **Cu**^**2+**^  < **Pb**^**2+**^ ions^54^. The adsorption capacity of carbon-based sorbent (graphene oxide) for hard ions (Na^+^ and Mg^2+^) is lower than for soft (Cd^2+^ and Pb^2+^) and borderline ions (Cu^2+^, Co^2+^, Zn^2+^, Ni^2+^) ions. This trend of the adsorption capacity agrees well with the trend presented in this study. Therefore, the adsorption capacity can be correlated to the metal ion characteristics such as the ionic radius, ionization potential, electronegativity, and covalent index and researchers can easily predict the adsorption performance.

It is worth to mention that LaFeO_3_/biochar composite offers an excellent adsorption capacity for Pb^2+^ ions, Table 3 summarizes a comparison of *q*_*m*_ values between the proposed sorbent in this study and recent reported sorbents in literature.

**Table 3:** A comparison of the adsorption capacity of LaFeO_3_/biochar for Pb^2+^ ions with literature.

Sorbent	*q*_*m*_ (mg/g)
CaTiO_3_ microspheres^35^	141.8
LaGdO_3_^36^	1.3
MnFe_2_O_4_-BiFeO_3_^37^	2277.0
MnFe_2_O_4_@Mn-Co oxide^56^	480.2
Biochar from Rice straw^57^	176.1
Biochar from banana peels^58^	247.1
Biochar from Sludge^59^	180.0
Biochar/Fe NPs^44^	17.3
LaFeO_3_/biochar^This work^	606.1

#### Real samples and interferences study

**Figure 5:**
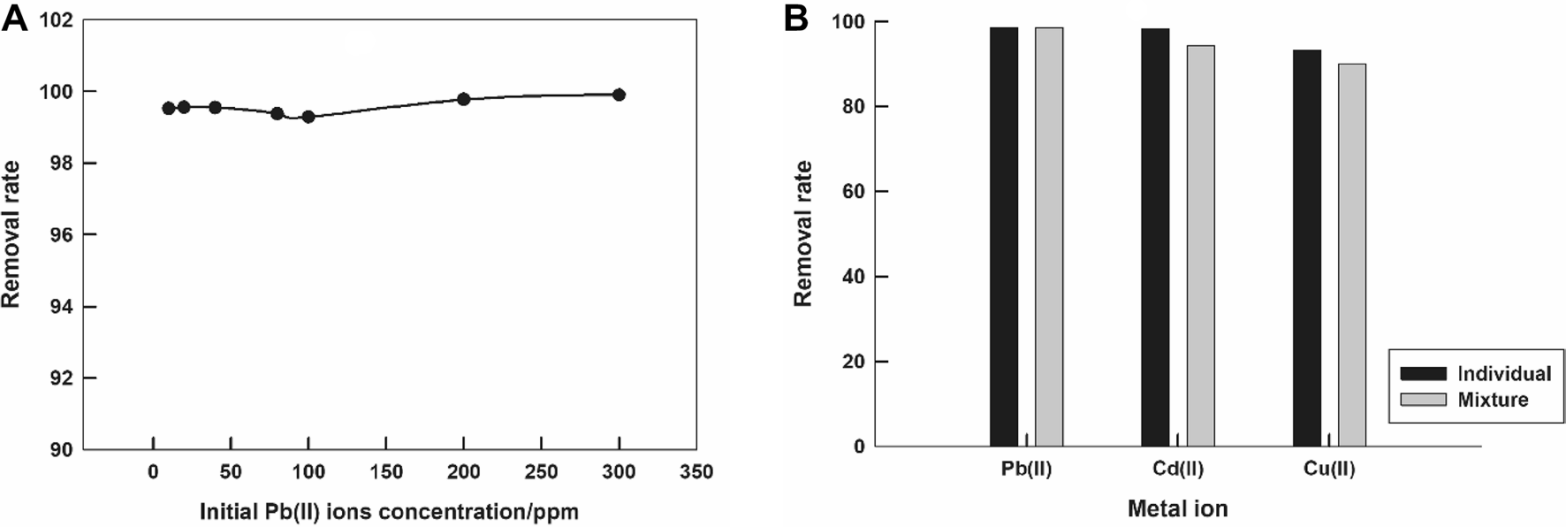
The dependence of the removal rate of Pb^2+^ ions by LaFeO_3_/biochar composite on the initial metal ion concentration in wastewater, pH = 7, contact time = 2 h, at room temperature, the inset represents Langmuir isotherm (**A**). The removal rate for different metal ions individually and in a ternary mixture (**B**).

The adsorption performance of LaFeO_3_/biochar composite for Pb^2+^ ions in wastewater (taken from a sewer) is examined. Figure 5A represents the variation of the removal rate with the initial Pb^2+^ ions concentration (10–300 ppm). It can be noticed that the removal rate is high (~ 99.5%) and independent of the initial concentration. According to Langmuir adsorption isotherm for the adsorption of Pb^2+^ ions on LaFeO_3_/biochar composite from wastewater, Figure S3, the calculated *q*_*m*_ and *K*_*L*_ values are 526.32 mg/g and 0.22 L/mg, respectively. As compared to the performance in distilled water, *q*_*m*_ = 606.06 mg/g, it is clear that LaFeO_3_/biochar composite maintains its excellent performance for the Pb^2+^ ions removal even in the presence of the real sample matrix effect.

In order to test the selectivity of the proposed sorbent for the studied metal ions, the removal rate values of Pb^2+^, Cd^2+^, and Cu^2+^ ions by LaFeO_3_/biochar composite, are calculated in a mixture containing equivalent concentrations of the three metal ions, and compared to the removal rate for each individual ion. Figure 5B shows a comparison between the removal rate values determined individually for each ion and in the ternary mixture of Pb^2+^, Cd^2+^, and Cu^2+^ ions. It can be noticed that the selectivity of LaFeO_3_/biochar composite for Pb^2+^ ions is excellent and unaffected by the presence of Cd^2+^, and Cu^2+^ ions. This finding is ascertained by the equal values of the removal rate for Pb^2+^ ions, determined individually and in the ternary mixture. While for Cd^2+^, and Cu^2+^ ions, the removal rate values, determined in the ternary mixture, decreased by 4% and 3.3%, respectively, which reflects the high selectivity of LaFeO_3_/biochar composite for Cd^2+^, and Cu^2+^ ions.

#### Regeneration and reuse

The sorbent, LaFeO_3_/biochar composite, can be regenerated by shaking it in 1% nitric acid solution for 24 h. This step is followed by filtration and drying of the sorbent before its reuse. The removal rate for Pb^2+^ ions by fresh and regenerated LaFeO_3_/biochar composite, after three cycles, are 98.2% and 95.2%, respectively, as shown in supplementary, Figure S4. The reduced adsorption ability using the regenerated sorbent is about 3%. It is concluded that the proposed sorbent can be successfully and easily regenerated and effectively reused for application in field use.

## Experimental

### Materials

Lanthanum (III) nitrate hexahydrate (99%), Iron (III) nitrate nonahydrate (98%), citric acid (98%), ammonium hydroxide solution (30–33%), nitric acid (69%), lead nitrate (99%), cadmium chloride hydrate (98%), and copper sulfate pentahydrate (98%) are bought from Sigma-Aldrich.

### Synthesis of LaFeO_3_/biochar composite

Banana peel (BP) is used as a source of cellulose; peels are washed with water, cut into small pieces and dried at 60 °C in an oven overnight. Finally, it is grounded before use. LaFeO_3_/biochar composite is prepared by the microwave-assisted citrate method. La(NO_3_)_3_.6H_2_O and Fe(NO_3_)_3_.9H_2_O are mixed in equimolar ratio, and dissolved in distilled water. Grounded BP is added to the aqueous metals ions solution, La^3+^ and Fe^3+^ ions, and allowed to shake for 24 h, to facilitates the adsorption of the metal ions on the cellulosic material. Then the pH of the mixture is adjusted at 8 using 1 mmol L^−1^ NH_4_OH and 1 mmol L^−1^ HNO_3_ solutions. Citric acid is then added in a molar amount twice that of the total metal ions. The mixed complex/cellulose suspension is evaporated and heated in the microwave (720 W) for 30 min. in a pulsed mode; 20-s on and 10-s off. The residual is ignited and turned into a fluffy powder, which is grounded and calcinated at 450 °C for 3 h to obtain LaFeO_3_/biochar composite^33^. All methods were performed in accordance with the relevant regulations of Cairo university.

### Adsorption experiment

The adsorption experiment is performed by adding 0.05 g of the sorbent, LaFeO_3_/biochar composite, to 25 mL of a given concentration of the pollutant metal ion solution (Pb^2+^, Cd^2+^, or Cu^2+^). The pH of the solution is previously adjusted at a given value (The investigated pH range is from 4 to 10). The adsorbate-sorbent suspension is shaken for a certain period that is extended from few minutes to a day. Then, the filtrate is separated from the solid material by using a nylon syringe filter, in order to determine the remaining concentration of the pollutant metal ion in the solution by the atomic absorption spectroscopy (NovAA 350).

The adsorption or removal rate is determined by the following equation:

7 $$Removal \,rate= \frac{{C}_{o}-{C}_{e}}{{C}_{o}} \times 100$$

The amount adsorbed of the metal ion, *q*, in mg/g is given by:

8 $$q= \frac{{C}_{ads \times {V}_{L}}}{m}$$

where *C*_*o*_, *C*_*e*_, and *C*_*ads*_ are initial, remaining, and adsorbed concentrations of the pollutant metal ion (mg/g), respectively. *V*_*L*_ is the volume of the adsorption solution (L), and *m* is the mass of the sorbent (g).

### Structural and surface characterization

Structural characterization is performed by Raman spectroscopy, Horiba labRAM HR evolution visible single spectrometer. The particle size distribution, examined by dynamic light scattering (DLS) method, is determined by the zeta seizer instrument (NanoSight NS500, Malvern Panalytical). Surface morphology is examined by scanning electron microscope (SEM), (JEOL JXA-840A). the surface area, pore size, and pore volume are calculated by Brunauer–Emmett–Teller (BET) method using N_2_ gas as an adsorbate at 77 K, and performed by Nova Touch, Quanta Chrome.

## Conclusion

LaFeO_3_/biochar composite is successively prepared by the cellulose-modified microwave-assisted citrate method at relatively low calcination temperature of 450 °C, as proved by the appearance of characteristic biochar bands, G, D, and 2D bands, and perovskite octahedral chemical shifts in Raman spectrum. Both rough microporous biochar and orthorhombic LaFeO_3_ nanoparticles. The prepared composite has excellent sorbent ability for Pb^2+^, Cd^2+^, and Cu^2+^ ions at pH ≥ 7, due to the electrostatic interactions between the positively charged metal ions and the negatively charge composite. At pH < 7, the removal ability is diminished due to the repulsion with the positively charged composite. However, for Pb^2+^ ions, the removal rate is unaffected and still high, due to the complex formation between Pb^2+^ ions and functional groups at the composite surface. The adsorption follows 2nd order kinetics, which suggests a chemisorption mode. The intraparticle-diffusion model is applied to the kinetic data, it is found that both surface and pore diffusion contribute equally to the rate-controlling step. Langmuir isotherms is applicable for the adsorption of Pb^2+^ ions, while Temkin isotherm fits best to the adsorption data of Cd^2+^, and Cu^2+^ ions. According to the metal ion characters, the interaction between Pb^2+^ ion and the proposed sorbent is due to the complex formation, while with Cd^2+^, and Cu^2+^ ions, the interaction is mainly electrostatic. LaFeO_3_/biochar composite can be easily regenerated and reused effectively, it has high selectivity to the studied metal ions and offers an excellent adsorption performance in real sample.

## Supplementary Information

Supplementary Information.

## Data Availability

Data supported the research findings is available upon request by contacting the corresponding author.
