# Supplementary material for: A correlation of the adsorption capacity of perovskite/biochar composite with the metal ion characteristics
Source: Sci Rep. 2023 Jun 10;13:9466. doi: 10.1038/s41598-023-36592-5 (PMC10257711; doi:10.1038/s41598-023-36592-5)
Supplement: Supplementary file 1 — Supplementary Information. [file 41598_2023_36592_MOESM1_ESM.docx]

|  |  |
| --- | --- |
|  |  |

**Figure S1:** **pseudo 1^st^ order (A, A’), and pseudo 2^nd^ order (B, B’) kinetic models for the adsorption of Cd^2+^ and Cu^2+^ ions on LaFeO_3_/biochar composite.**

| **Langmuir isotherm** | **Freundlich isotherm** | **Temkin isotherm** |
| --- | --- | --- |
|  |  |  |
|  |  |  |
|  |  |  |

**Figure S2: Langmuir (A, A’, A’’), Freundlich (B, B’, B’’), and Temkin (C, C’, C’’) adsorption isotherm for the adsorption of Pb^2+^, Cd^2+^, and Cu^2+^ ions by LaFeO_3_/biochar composite, respectively.**

**
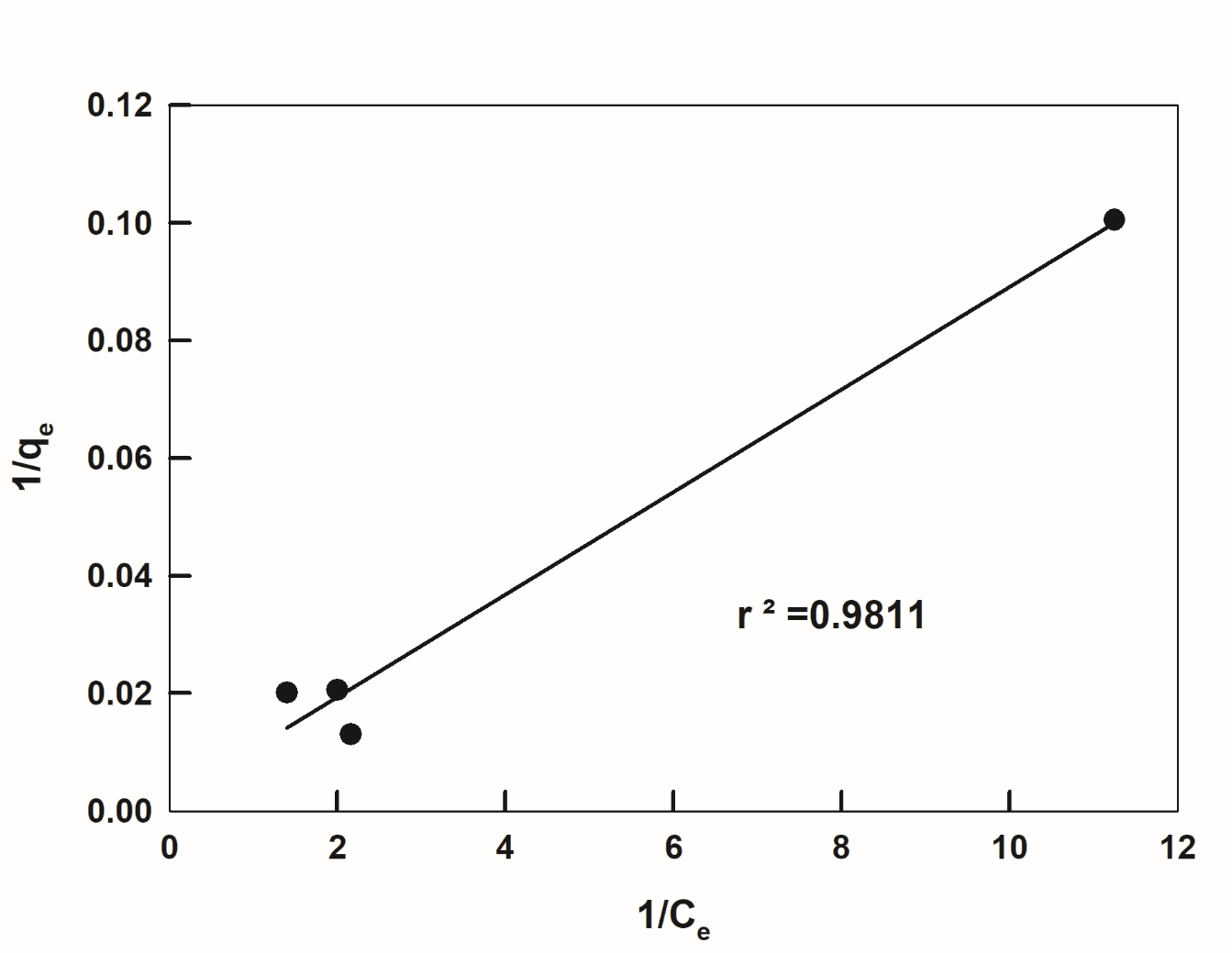
**

**Figure S3: Langmuir adsorption isotherm for the adsorption of Pb^2+^ ions on LaFeO_3_/biochar composite from wastewater.**

**
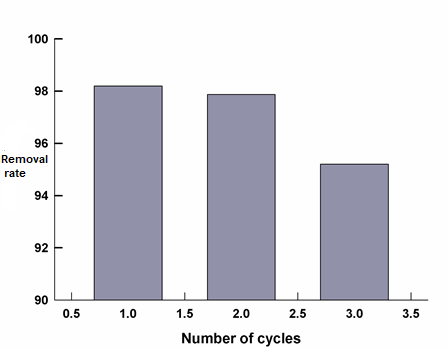
**

**Figure S4: The variation of the removal % of LaFeO_3_/biochar composite for Pb^2+^ ions with the number of regeneration and reuse cycles.**

**Table S1: Content of wastewater used for the real sample.**

| mg L^-1^ | | | | | | | | | | | | |
| --- | --- | --- | --- | --- | --- | --- | --- | --- | --- | --- | --- | --- |
| NH_4_ | NO_3_ | P | Fe | Mn | Zn | Cu | B | Cd | Co | Cr | Ni | Pb |
| 4.27 | 14.63 | 0.288 | 0.036 | 0.007 | 0.371 | 0.057 | 0.18 | 0 | nd | 0.001 | 0.004 | 0.007 |

| pH | TDS (ppm) | EC  dSm^-1^ | Anions meq L^-1^ | | | | Cations meq L^-1^ | | | | SAR |
| --- | --- | --- | --- | --- | --- | --- | --- | --- | --- | --- | --- |
|  |  |  | CO_3_^-^ | HCO_3_^-^ | Cl^-^ | SO_4_^--^ | Ca^++^ | Mg^++^ | Na^+^ | K^+^ |  |
| 6.9 | 2508.8 | 3.92 | 0 | 3.02 | 23.39 | 9.14 | 8.92 | 6.67 | 19.13 | 0.84 | 6.85 |
